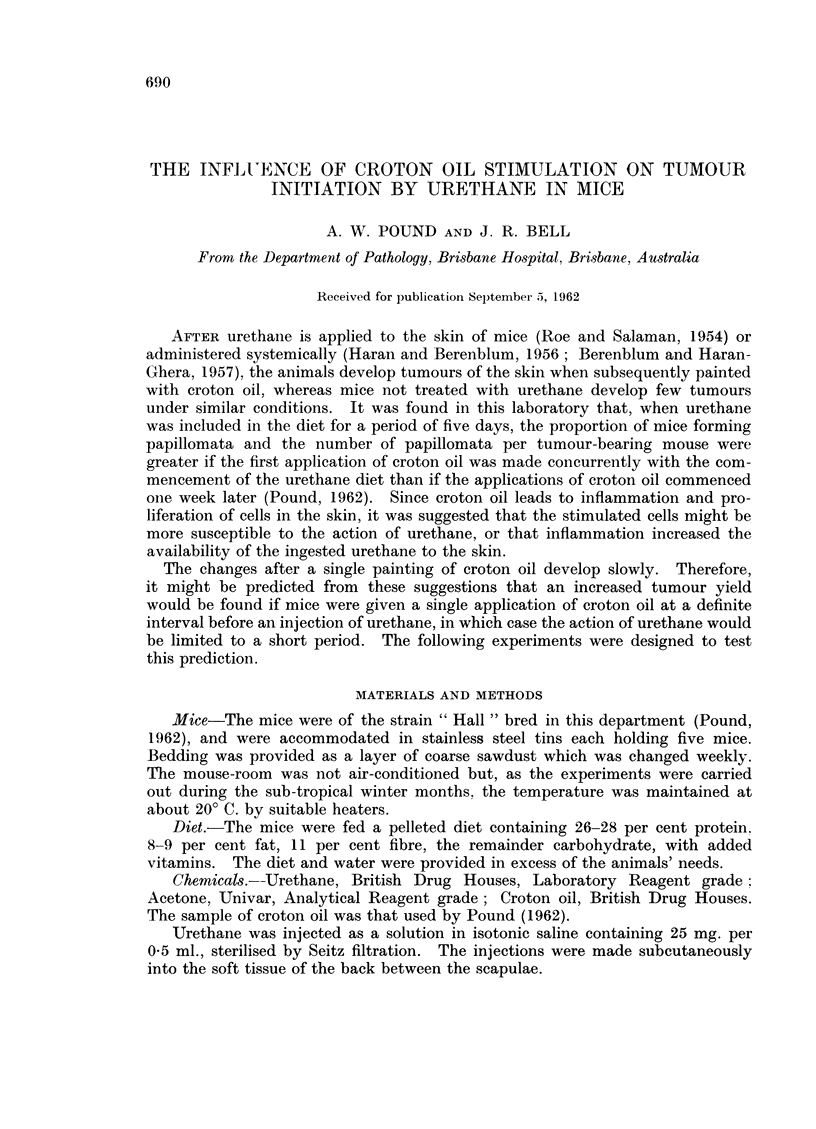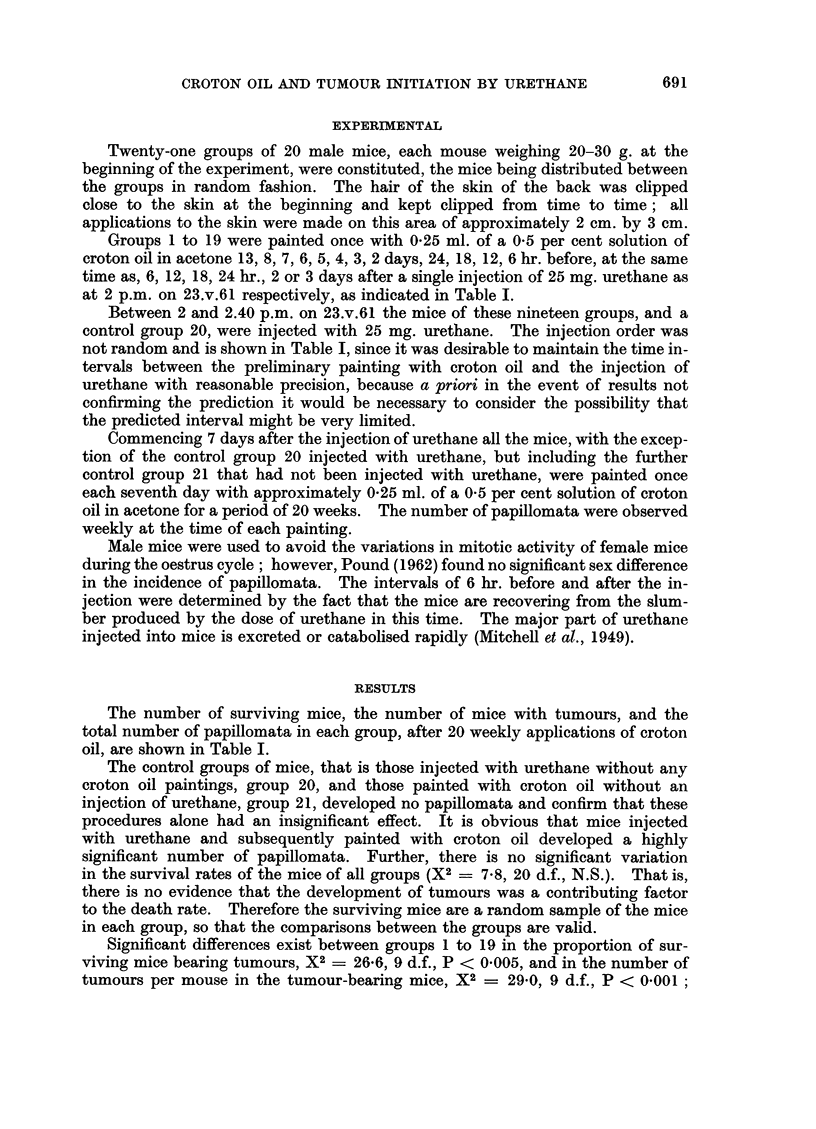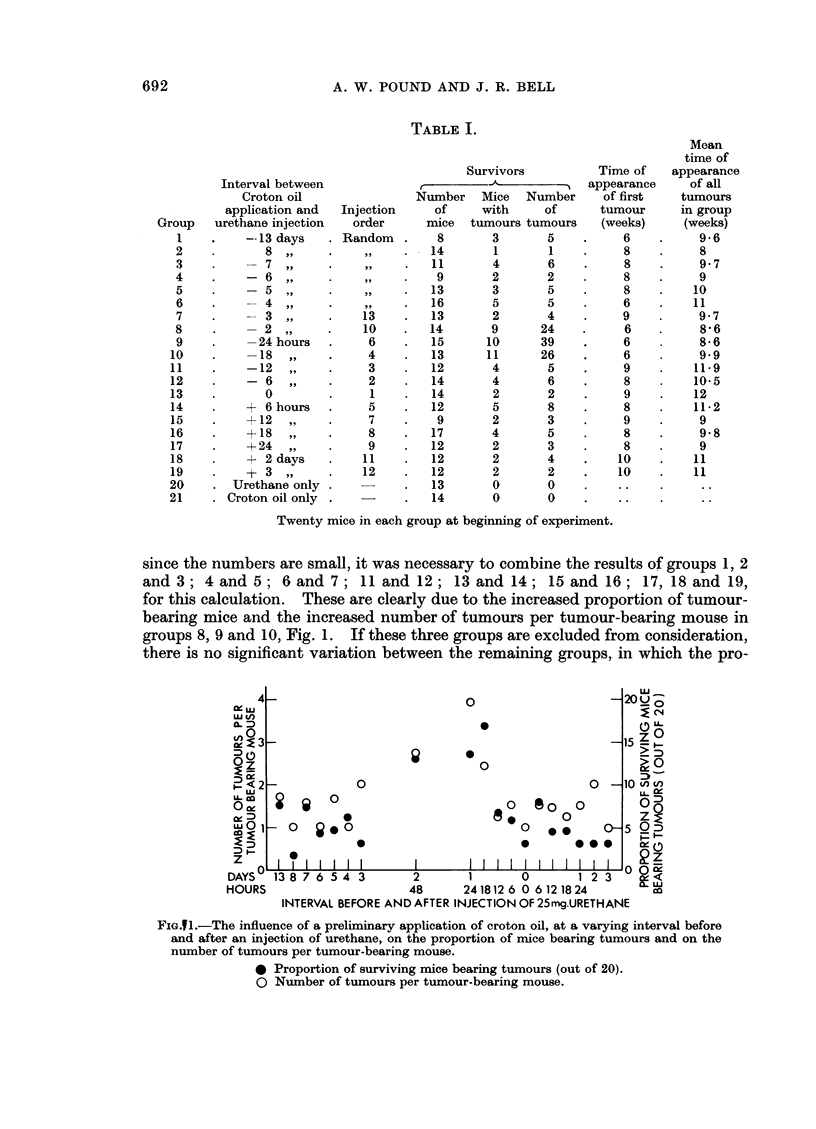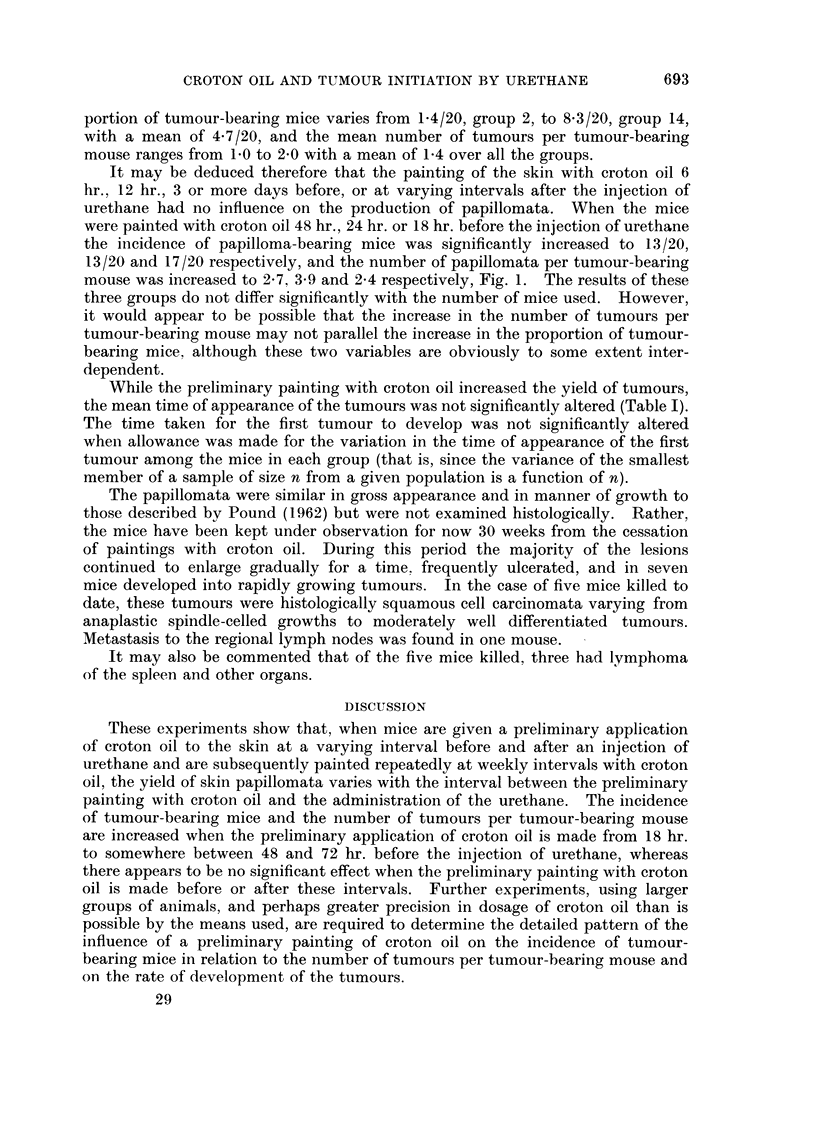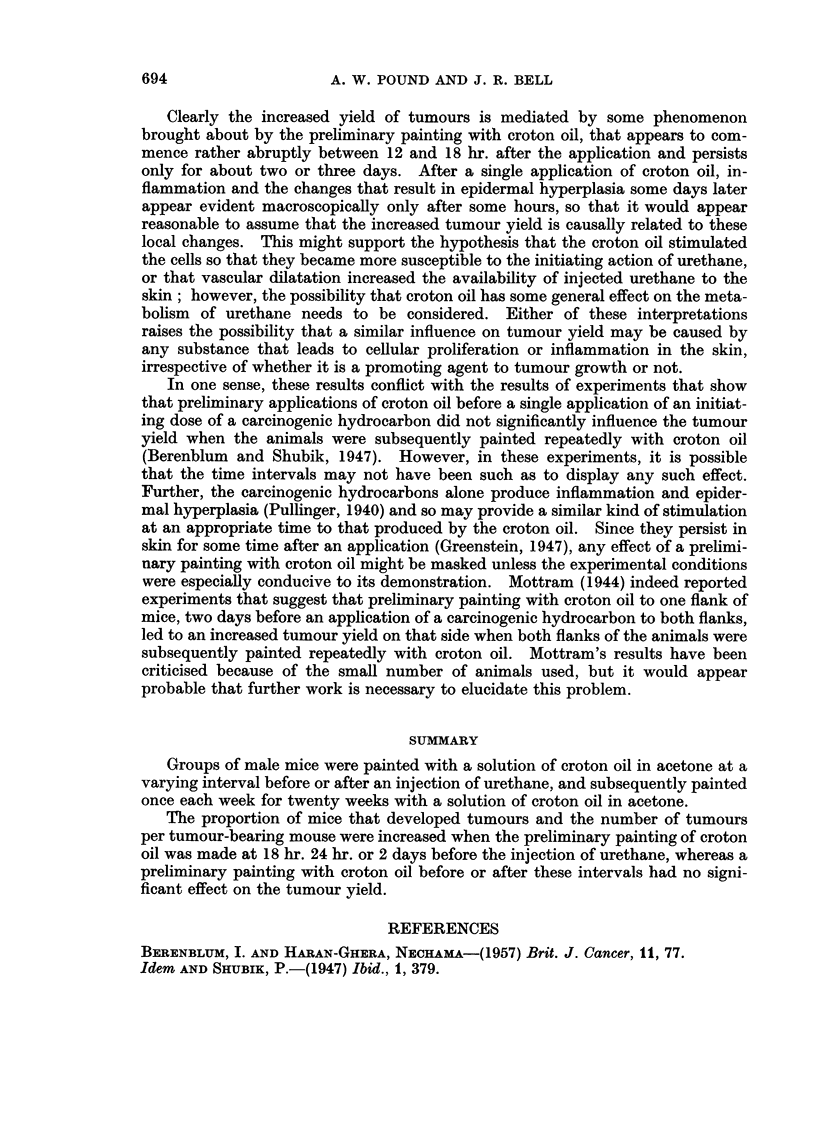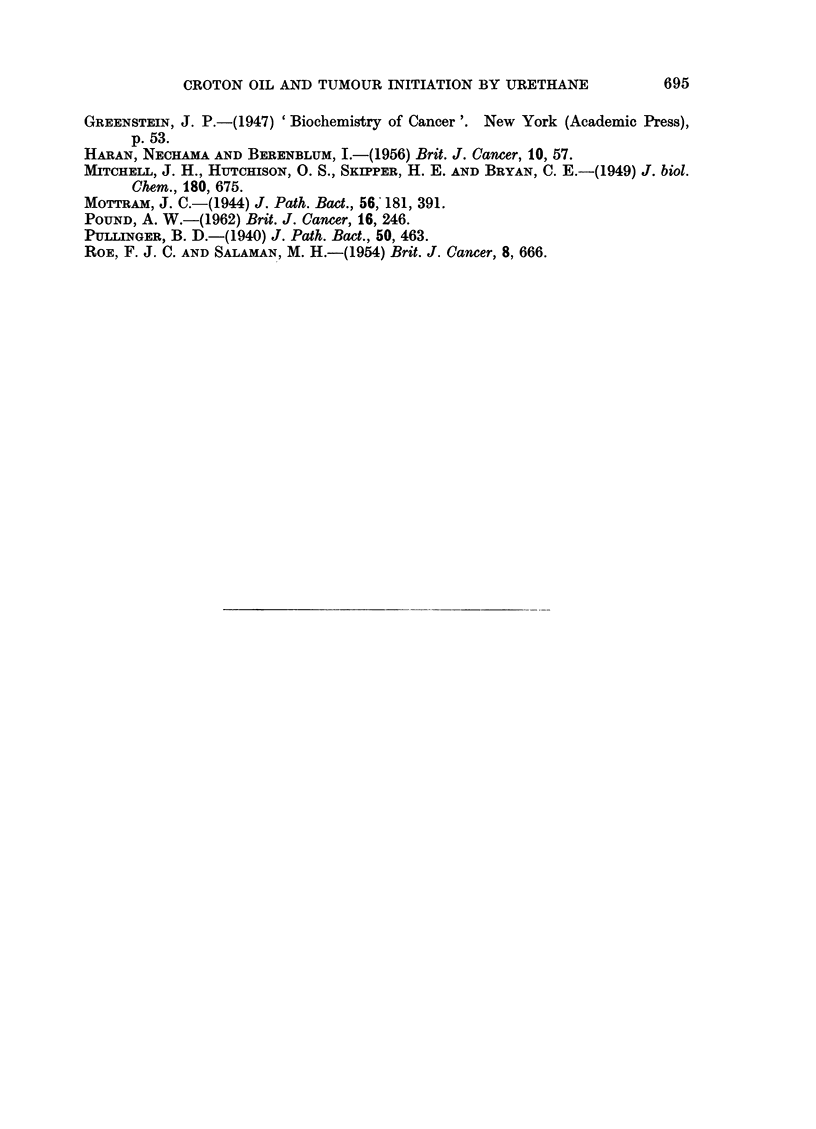# The Influence of Croton Oil Stimulation on Tumour Initiation by Urethane in Mice

**DOI:** 10.1038/bjc.1962.79

**Published:** 1962-12

**Authors:** A. W. Pound, J. R. Bell


### THE INFLUENCE OF CROTON OIL STIMULATION ON TUMOUR INITIATION BY URETHANE IN MICE

A. W. POUND AND J. R. BELL

*From the Department of Pathology, Brisbane Hospital, Brisbane, Australia*

Received for publication September 5, 1962

AFTER urethane is applied to the skin of mice (Roe and Salaman, 1954) or administered systemically (Haran and Berenblum, 1956 ; Berenblum and Haran-Ghera, 1957), the animals develop tumours of the skin when subsequently painted with croton oil, whereas mice not treated with urethane develop few tumours under similar conditions. It was found in this laboratory that, when urethane was included in the diet for a period of five days, the proportion of mice forming papillomata and the number of papillomata per tumour-bearing mouse were greater if the first application of croton oil was made concurrently with the commencement of the urethane diet than if the applications of croton oil commenced one week later (Pound, 1962). Since croton oil leads to inflammation and proliferation of cells in the skin, it was suggested that the stimulated cells might be more susceptible to the action of urethane, or that inflammation increased the availability of the ingested urethane to the skin.

The changes after a single painting of croton oil develop slowly. Therefore, it might be predicted from these suggestions that an increased tumour yield would be found if mice were given a single application of croton oil at a definite interval before an injection of urethane, in which case the action of urethane would be limited to a short period. The following experiments were designed to test this prediction.

### MATERIALS AND METHODS

*Mice*—The mice were of the strain " Hall " bred in this department (Pound, 1962), and were accommodated in stainless steel tins each holding five mice. Bedding was provided as a layer of coarse sawdust which was changed weekly. The mouse-room was not air-conditioned but, as the experiments were carried out during the sub-tropical winter months, the temperature was maintained at about 20° C. by suitable heaters.

*Diet.*—The mice were fed a pelleted diet containing 26–28 per cent protein, 8–9 per cent fat, 11 per cent fibre, the remainder carbohydrate, with added vitamins. The diet and water were provided in excess of the animals' needs.

*Chemicals.*—Urethane, British Drug Houses, Laboratory Reagent grade ; Acetone, Univar, Analytical Reagent grade ; Croton oil, British Drug Houses. The sample of croton oil was that used by Pound (1962).

Urethane was injected as a solution in isotonic saline containing 25 mg. per 0·5 ml., sterilised by Seitz filtration. The injections were made subcutaneously into the soft tissue of the back between the scapulae.

### EXPERIMENTAL

Twenty-one groups of 20 male mice, each mouse weighing 20–30 g. at the beginning of the experiment, were constituted, the mice being distributed between the groups in random fashion. The hair of the skin of the back was clipped close to the skin at the beginning and kept clipped from time to time ; all applications to the skin were made on this area of approximately 2 cm. by 3 cm.

Groups 1 to 19 were painted once with 0·25 ml. of a 0·5 per cent solution of croton oil in acetone 13, 8, 7, 6, 5, 4, 3, 2 days, 24, 18, 12, 6 hr. before, at the same time as, 6, 12, 18, 24 hr., 2 or 3 days after a single injection of 25 mg. urethane as at 2 p.m. on 23.v.61 respectively, as indicated in Table I.

Between 2 and 2.40 p.m. on 23.v.61 the mice of these nineteen groups, and a control group 20, were injected with 25 mg. urethane. The injection order was not random and is shown in Table I, since it was desirable to maintain the time intervals between the preliminary painting with croton oil and the injection of urethane with reasonable precision, because *a priori* in the event of results not confirming the prediction it would be necessary to consider the possibility that the predicted interval might be very limited.

Commencing 7 days after the injection of urethane all the mice, with the exception of the control group 20 injected with urethane, but including the further control group 21 that had not been injected with urethane, were painted once each seventh day with approximately 0·25 ml. of a 0·5 per cent solution of croton oil in acetone for a period of 20 weeks. The number of papillomata were observed weekly at the time of each painting.

Male mice were used to avoid the variations in mitotic activity of female mice during the oestrus cycle ; however, Pound (1962) found no significant sex difference in the incidence of papillomata. The intervals of 6 hr. before and after the injection were determined by the fact that the mice are recovering from the slumber produced by the dose of urethane in this time. The major part of urethane injected into mice is excreted or catabolised rapidly (Mitchell *et al.*, 1949).

### RESULTS

The number of surviving mice, the number of mice with tumours, and the total number of papillomata in each group, after 20 weekly applications of croton oil, are shown in Table I.

The control groups of mice, that is those injected with urethane without any croton oil paintings, group 20, and those painted with croton oil without an injection of urethane, group 21, developed no papillomata and confirm that these procedures alone had an insignificant effect. It is obvious that mice injected with urethane and subsequently painted with croton oil developed a highly significant number of papillomata. Further, there is no significant variation in the survival rates of the mice of all groups ($X^2 = 7{\cdot}8$, 20 d.f., N.S.). That is, there is no evidence that the development of tumours was a contributing factor to the death rate. Therefore the surviving mice are a random sample of the mice in each group, so that the comparisons between the groups are valid.

Significant differences exist between groups 1 to 19 in the proportion of surviving mice bearing tumours, $X^2 = 26{\cdot}6$, 9 d.f., $P < 0{\cdot}005$, and in the number of tumours per mouse in the tumour-bearing mice, $X^2 = 29{\cdot}0$, 9 d.f., $P < 0{\cdot}001$ ;

TABLE I.

| Group | Interval between Croton oil application and urethane injection | Injection order | Survivors: Number of mice | Survivors: Mice with tumours | Survivors: Number of tumours | Time of appearance of first tumour (weeks) | Mean time of appearance of all tumours in group (weeks) |
|---|---|---|---|---|---|---|---|
| 1 | −13 days | Random | 8 | 3 | 5 | 6 | 9·6 |
| 2 | 8 ,, | ,, | 14 | 1 | 1 | 8 | 8 |
| 3 | − 7 ,, | ,, | 11 | 4 | 6 | 8 | 9·7 |
| 4 | − 6 ,, | ,, | 9 | 2 | 2 | 8 | 9 |
| 5 | − 5 ,, | ,, | 13 | 3 | 5 | 8 | 10 |
| 6 | − 4 ,, | ,, | 16 | 5 | 5 | 6 | 11 |
| 7 | − 3 ,, | 13 | 13 | 2 | 4 | 9 | 9·7 |
| 8 | − 2 ,, | 10 | 14 | 9 | 24 | 6 | 8·6 |
| 9 | −24 hours | 6 | 15 | 10 | 39 | 6 | 8·6 |
| 10 | −18 ,, | 4 | 13 | 11 | 26 | 6 | 9·9 |
| 11 | −12 ,, | 3 | 12 | 4 | 5 | 9 | 11·9 |
| 12 | − 6 ,, | 2 | 14 | 4 | 6 | 8 | 10·5 |
| 13 | 0 | 1 | 14 | 2 | 2 | 9 | 12 |
| 14 | + 6 hours | 5 | 12 | 5 | 8 | 8 | 11·2 |
| 15 | +12 ,, | 7 | 9 | 2 | 3 | 9 | 9 |
| 16 | +18 ,, | 8 | 17 | 4 | 5 | 8 | 9·8 |
| 17 | +24 ,, | 9 | 12 | 2 | 3 | 8 | 9 |
| 18 | + 2 days | 11 | 12 | 2 | 4 | 10 | 11 |
| 19 | + 3 ,, | 12 | 12 | 2 | 2 | 10 | 11 |
| 20 | Urethane only | — | 13 | 0 | 0 | .. | .. |
| 21 | Croton oil only | — | 14 | 0 | 0 | .. | .. |

Twenty mice in each group at beginning of experiment.

since the numbers are small, it was necessary to combine the results of groups 1, 2 and 3 ; 4 and 5 ; 6 and 7 ; 11 and 12 ; 13 and 14 ; 15 and 16 ; 17, 18 and 19, for this calculation. These are clearly due to the increased proportion of tumour-bearing mice and the increased number of tumours per tumour-bearing mouse in groups 8, 9 and 10, Fig. 1. If these three groups are excluded from consideration, there is no significant variation between the remaining groups, in which the pro-

FIG. 1.—The influence of a preliminary application of croton oil, at a varying interval before and after an injection of urethane, on the proportion of mice bearing tumours and on the number of tumours per tumour-bearing mouse.

● Proportion of surviving mice bearing tumours (out of 20).
○ Number of tumours per tumour-bearing mouse.

portion of tumour-bearing mice varies from 1·4/20, group 2, to 8·3/20, group 14, with a mean of 4·7/20, and the mean number of tumours per tumour-bearing mouse ranges from 1·0 to 2·0 with a mean of 1·4 over all the groups.

It may be deduced therefore that the painting of the skin with croton oil 6 hr., 12 hr., 3 or more days before, or at varying intervals after the injection of urethane had no influence on the production of papillomata. When the mice were painted with croton oil 48 hr., 24 hr. or 18 hr. before the injection of urethane the incidence of papilloma-bearing mice was significantly increased to 13/20, 13/20 and 17/20 respectively, and the number of papillomata per tumour-bearing mouse was increased to 2·7, 3·9 and 2·4 respectively, Fig. 1. The results of these three groups do not differ significantly with the number of mice used. However, it would appear to be possible that the increase in the number of tumours per tumour-bearing mouse may not parallel the increase in the proportion of tumour-bearing mice, although these two variables are obviously to some extent inter-dependent.

While the preliminary painting with croton oil increased the yield of tumours, the mean time of appearance of the tumours was not significantly altered (Table I). The time taken for the first tumour to develop was not significantly altered when allowance was made for the variation in the time of appearance of the first tumour among the mice in each group (that is, since the variance of the smallest member of a sample of size $n$ from a given population is a function of $n$).

The papillomata were similar in gross appearance and in manner of growth to those described by Pound (1962) but were not examined histologically. Rather, the mice have been kept under observation for now 30 weeks from the cessation of paintings with croton oil. During this period the majority of the lesions continued to enlarge gradually for a time, frequently ulcerated, and in seven mice developed into rapidly growing tumours. In the case of five mice killed to date, these tumours were histologically squamous cell carcinomata varying from anaplastic spindle-celled growths to moderately well differentiated tumours. Metastasis to the regional lymph nodes was found in one mouse.

It may also be commented that of the five mice killed, three had lymphoma of the spleen and other organs.

### DISCUSSION

These experiments show that, when mice are given a preliminary application of croton oil to the skin at a varying interval before and after an injection of urethane and are subsequently painted repeatedly at weekly intervals with croton oil, the yield of skin papillomata varies with the interval between the preliminary painting with croton oil and the administration of the urethane. The incidence of tumour-bearing mice and the number of tumours per tumour-bearing mouse are increased when the preliminary application of croton oil is made from 18 hr. to somewhere between 48 and 72 hr. before the injection of urethane, whereas there appears to be no significant effect when the preliminary painting with croton oil is made before or after these intervals. Further experiments, using larger groups of animals, and perhaps greater precision in dosage of croton oil than is possible by the means used, are required to determine the detailed pattern of the influence of a preliminary painting of croton oil on the incidence of tumour-bearing mice in relation to the number of tumours per tumour-bearing mouse and on the rate of development of the tumours.

Clearly the increased yield of tumours is mediated by some phenomenon brought about by the preliminary painting with croton oil, that appears to commence rather abruptly between 12 and 18 hr. after the application and persists only for about two or three days. After a single application of croton oil, inflammation and the changes that result in epidermal hyperplasia some days later appear evident macroscopically only after some hours, so that it would appear reasonable to assume that the increased tumour yield is causally related to these local changes. This might support the hypothesis that the croton oil stimulated the cells so that they became more susceptible to the initiating action of urethane, or that vascular dilatation increased the availability of injected urethane to the skin ; however, the possibility that croton oil has some general effect on the metabolism of urethane needs to be considered. Either of these interpretations raises the possibility that a similar influence on tumour yield may be caused by any substance that leads to cellular proliferation or inflammation in the skin, irrespective of whether it is a promoting agent to tumour growth or not.

In one sense, these results conflict with the results of experiments that show that preliminary applications of croton oil before a single application of an initiating dose of a carcinogenic hydrocarbon did not significantly influence the tumour yield when the animals were subsequently painted repeatedly with croton oil (Berenblum and Shubik, 1947). However, in these experiments, it is possible that the time intervals may not have been such as to display any such effect. Further, the carcinogenic hydrocarbons alone produce inflammation and epidermal hyperplasia (Pullinger, 1940) and so may provide a similar kind of stimulation at an appropriate time to that produced by the croton oil. Since they persist in skin for some time after an application (Greenstein, 1947), any effect of a preliminary painting with croton oil might be masked unless the experimental conditions were especially conducive to its demonstration. Mottram (1944) indeed reported experiments that suggest that preliminary painting with croton oil to one flank of mice, two days before an application of a carcinogenic hydrocarbon to both flanks, led to an increased tumour yield on that side when both flanks of the animals were subsequently painted repeatedly with croton oil. Mottram's results have been criticised because of the small number of animals used, but it would appear probable that further work is necessary to elucidate this problem.

### SUMMARY

Groups of male mice were painted with a solution of croton oil in acetone at a varying interval before or after an injection of urethane, and subsequently painted once each week for twenty weeks with a solution of croton oil in acetone.

The proportion of mice that developed tumours and the number of tumours per tumour-bearing mouse were increased when the preliminary painting of croton oil was made at 18 hr. 24 hr. or 2 days before the injection of urethane, whereas a preliminary painting with croton oil before or after these intervals had no significant effect on the tumour yield.

### REFERENCES

BERENBLUM, I. AND HARAN-GHERA, NECHAMA—(1957) *Brit. J. Cancer*, **11**, 77.
*Idem* AND SHUBIK, P.—(1947) *Ibid.*, **1**, 379.

GREENSTEIN, J. P.—(1947) ' Biochemistry of Cancer '. New York (Academic Press), p. 53.
HARAN, NECHAMA AND BERENBLUM, I.—(1956) *Brit. J. Cancer*, **10**, 57.
MITCHELL, J. H., HUTCHISON, O. S., SKIPPER, H. E. AND BRYAN, C. E.—(1949) *J. biol. Chem.*, **180**, 675.
MOTTRAM, J. C.—(1944) *J. Path. Bact.*, **56**, 181, 391.
POUND, A. W.—(1962) *Brit. J. Cancer*, **16**, 246.
PULLINGER, B. D.—(1940) *J. Path. Bact.*, **50**, 463.
ROE, F. J. C. AND SALAMAN, M. H.—(1954) *Brit. J. Cancer*, **8**, 666.